# Determinants of Measles Vaccine Hesitancy among Sudanese Parents in Khartoum State, Sudan: A Cross-Sectional Study

**DOI:** 10.3390/vaccines10010006

**Published:** 2021-12-22

**Authors:** Majdi M. Sabahelzain, Mohamed Moukhyer, Hans Bosma, Bart van den Borne

**Affiliations:** 1 Department of Public Health, School of Health Sciences, Ahfad University for Women, Omdurman P.O. Box 167, Sudan; 2 Department of Health Promotion, Care and Public Health Research Institute (CAPHRI), Maastricht University, 6211 LK Maastricht, The Netherlands; b.vdborne@maastrichtuniversity.nl; 3 Education Development and Quality Unit, College of Applied Medical Sciences, Jazan University, Jazan 45142, Saudi Arabia; moukhyer@hotmail.com; 4 Public Health Programmes, School of Medicine, University of Limerick, V94 T9PX Limerick, Ireland; 5 Department of Social Medicine, Care and Public Health Research Institute (CAPHRI), Maastricht University, 6211 LK Maastricht, The Netherlands; hans.bosma@maastrichtuniversity.nl

**Keywords:** measles vaccine, vaccine hesitancy, PACV, Sudan, vaccine access

## Abstract

Determinants of vaccine hesitancy are not yet well understood. This study aims to assess measles vaccine hesitancy and characterize its determinants among Sudanese parents in Omdurman in Khartoum State. A community-based cross-sectional quantitative study was conducted in Khartoum State in February 2019. The Parent Attitudes about Childhood Vaccination (PACV) was used to measure measles vaccine hesitancy. Questions about the sociodemographic characteristics of the family, the perception of the parents about the measles vaccine, and the parental exposure to information were asked. Proportions of vaccine hesitancy and coefficients of linear regression were computed. Five hundred parents were recruited for the study. We found that a significant proportion of participants (about 1 in 5 parents) had hesitations regarding the measles vaccine. Significant predictors of measles vaccine hesitancy were parental exposure to anti-vaccination information or materials (β = −0.478, *p*-value < 0.001), the parents’ perception of the effectiveness of measles vaccines (β = 0.093, *p*-value = 0.020), the age of the mother (β = 0.112, *p*-value = 0.017), the birth rank of the child (β = −0.116, *p*-value = 0.015), and the total number of the children in the family (β = 0.098, *p*-value = 0.013). Vaccination access issues were the common justification for parental vaccination hesitancy. Our findings indicate that investment in vaccine communication as well as addressing access issues might be an effective intervention for improving measles vaccine acceptance and, ultimately, measles vaccine coverage.

## 1. Introduction

Vaccine hesitancy is one of the main predictors of low vaccine acceptance and demand worldwide [1,2,3]. As vaccine hesitancy is well investigated in high-income countries, reports show that vaccine hesitancy is increasing in these areas, posing a growing threat to global health [4,5,6,7]. As defined by the WHO Strategic Advisory Group of Experts on vaccine hesitancy (SAGE), vaccine hesitancy is “delay in acceptance or refusal of vaccines despite availability of vaccination services. Vaccine hesitancy is complex and context-specific, varying across time, place, and vaccines”. Additionally, as part of its mission, SAGE developed two models of determinants of vaccine hesitancy; the simplest one is the “3Cs” model, which includes complacency (perceived risks of vaccine-preventable diseases are low and no vaccines are needed), convenience (access issues and constraints), and confidence (i.e., level of trust in a vaccine), and the more comprehensive “Working Group Determinants of Vaccine Hesitancy Matrix”, which characterizes the complexity of the contextual, individual, and group, and vaccine/vaccination-specific influences [3,8].

The WHO reported that measles cases rose by 300% in the first quarter of 2019, compared to a similar period in 2018. Many countries around the world are suffering from measles outbreaks; four European counties have lost their measles elimination status, including Albania, the Czech Republic, Greece, and the United Kingdom [7,9]. Although the benefits of strong immunization programs are clear, suboptimal coverage exists among specific populations in certain countries in low- and middle-income countries (LMICs). Studies’ findings have attributed this suboptimal coverage to access and acceptance issues, low vaccine investments, and humanitarian crises [10,11,12].

Despite its public health and economic consequences, vaccine hesitancy and acceptance have not been yet well studied in LMICs, where the socioeconomic and sociocultural contexts differ; hence, possibly different interventions are needed [2,13,14,15]. In order to develop better interventions, more information is needed about the predictors of hesitancy. Recent findings underscore that lower vaccination coverage was more common among people with higher socioeconomic status in 10 upper-middle-income countries [16]. In LMICs, however, low uptake of childhood vaccination is associated with different determinants, including the behavioral and social factors of parents [17,18,19,20,21,22,23,24,25,26].

Sudan is one of the countries that has experienced measles outbreaks in the past two years. There were 4978 confirmed cases in 2018, an increase of 649% compared to the number of measles cases in 2017 [7]. Furthermore, the national vaccination coverage is suboptimal for the first and the second dose of the measles-containing vaccine (88% and 72%, respectively) [27]. Additional risk factors contributing to the measles burden in Sudan include political and economic instability, as they affect directly the nutritional status of the at-risk groups, as well as the difficulty of vaccinating children in conflict areas in Darfur, South Kordofan, and the Blue Nile states [28].

A recent study conducted among immunization experts and vaccine providers in Sudan showed that measles vaccine hesitancy exists in Sudan and is caused by many drivers, including religious and cultural drivers, beliefs about prevention and treatment, as well as causes related to the measles vaccine program [29]. To further inform the development of relevant policies and interventions, this study aimed to assess the measles vaccine hesitancy and characterize the determinants of hesitancy among Sudanese parents in Omdurman in Khartoum State.

## 2. Materials and Methods

### 2.1. Study Design

A community-based cross-sectional quantitative study was conducted in two areas in the Omdurman locality in Khartoum State in February 2019. These two areas were selected for the study because they reflect typical social and cultural groups in Sudan. Additionally, being in an urban setting provides relatively high exposure to different vaccination and communication campaigns. Furthermore, to assess vaccine hesitancy, the availability of vaccination services is a prerequisite for the study, which applies to the Omdurman locality. Data were collected using a written questionnaire. The questionnaires were completed either in the presence of an interviewer or administered by an interviewer.

### 2.2. Population and Sampling

#### 2.2.1. Population

The study population included parents/guardians with a child aged 2–3 years. To ensure that people from various sociocultural and socioeconomic backgrounds (i.e., education and wealth level) were included in this study, we performed a stratified sampling technique to collect data from parents/caregivers in the two different urban districts in Omdurman, Alsharafia (Wad Nubawi’s administrative unit) and Abo Saaeed (Abo Saaeed’s administrative unit). 

#### 2.2.2. Sampling

Either mothers or fathers were eligible for participation. If there was more than one child in the same age range in the family, the parents/guardians were asked to answer about only the youngest one to reduce recall bias. If both mother and father were available, they were asked to choose one of themselves to fill in the questionnaire.

This study is part of a larger research project about measles vaccine hesitancy in Sudan [29,30]. The sample size was calculated for the whole research project as follows: A power analysis for the association between measles vaccine hesitancy and the measles vaccination status (outcome) showed that at least 386 participants were needed to yield an 80% power to detect an odds ratio of 1.7 at alpha level (5%). Assuming the prevalence of the outcome, the measles vaccination status among the exposed group (hesitant parents) was 50% [31]. To cover for possible dropouts due to missing information on the crucial questions, 500 participants (parents/caregivers) were recruited for the study. Parents/caregivers were selected using consecutive sampling; every parent/caregiver meeting the criteria of inclusion was selected until the required sample size was achieved.

### 2.3. Data Collection

Data were collected using a pretested questionnaire. The dependent variable was the measles vaccine hesitancy, which was measured using the Parent Attitudes about Childhood Vaccination (PACV) scale. The independent variables in this study were the sociodemographic characteristics of the family that included the mother’s education, which was measured at four levels as described by the ministry of education, including none (not attended any formal or nonformal education), primary (this level lasts eight years from Grade 1 to Grade 8), secondary (ages 14 to 16 can attend secondary education, which lasts three years), and university level (this includes obtaining diploma, bachelor’s, and postgraduate degrees); the mother’s employment, which was measured at two levels (housewife, which is defined by the International Labor Organization as an unemployed mother who does housekeeping functions, and employed mother); income level of the family, which was self-ranked by the study participants on three levels (high, medium, and low); number of children who were aged <5 years in the family; and the rank of the child who was aged 2–3 years among his/her siblings (measured as (1st, 2nd, 3rd, and 4th and above). Additionally, questions about the parental exposure to either pro- and anti-vaccine information/materials or campaigns were asked (with a Yes/No answer). Additionally, the questionnaire included questions about the reasons behind the measles vaccine hesitancy, which were asked either when vaccine-hesitant respondents reported directly that they partially vaccinated or refused to vaccinate their children with the measles vaccine or when we found on the vaccination card the measles vaccination was missing or that only a single dose of measles vaccine was recorded.

#### Hesitancy Measurement Tool

In this study, we used the Parent Attitudes about Childhood Vaccination (PACV) to measure measles vaccine hesitancy as the dependent variable. PACV has been validated in Sudan [30]. The PACV includes 15 items categorized in three domains: immunization behavior (Items 1 and 2), perceived safety and efficacy (items 7–10), and general attitudes and trust (items 3–6 and 11–15).

In its original English version that was used in the USA, Cronbach alpha coefficients for the 3 sub-domain scales were 0.74, 0.84, and 0.74, respectively [32,33]. This scale was evaluated in many countries [20,21], including Sudan (The Cronbach’s alpha computed for Q3–Q15 was 0.62) [30]. Item scores for the total scale were summed to a total score ranging from 0 to 30. Parents with children who answered “don’t know” in the abovementioned behavior items were considered missing data because this response likely reflected poor recall rather than immunization hesitancy, as suggested by other studies. The total raw score was converted to a 0–100 scale [32,33,34,35].

### 2.4. Data Analysis

Data analysis was performed using Statistical Package for Social Sciences (SPSS) (version 24). The sociodemographic characteristics of the family, children’s vaccination status, and parents’ exposure to vaccination information/materials were presented in a table. Additionally, frequencies of the PACV items were calculated. Multivariate linear regression analysis was run to identify the factors predicting the dependent variable, measles vaccine hesitancy (PACV). A *p*-value of less than 0.05 was considered statistically significant. Reasons behind hesitancy given by measles vaccine-hesitant respondents (i.e., quotes) were matched to the categories of the “3Cs” model: confidence issues (concerns about the safety or efficacy of the vaccine, previous bad experiences, or preference for alternative health approaches), convenience (access issues), or complacency (perceptions that the vaccine was unimportant or unnecessary). 

## 3. Results

### 3.1. Sociodemographic Characteristics of the Family, Children’s Vaccination Status, and Parents’ Exposure to Vaccination Information/Materials

Data were collected from 500 parents/caregivers. As described in Table 1, the majority of the participants were mothers (87.2%) between the ages of 20 and 47 (M = 31.15, SD = 5.74) and housewives (74.6%), and 50% had a university education and postgraduate level. Most of the participants had two children who were aged less than five years (45.6%), the majority were fully vaccinated with the measles vaccine (87.2%). More than three-quarters of the participants (77.4%) reported that they have been exposed to pro-vaccination information or materials. However, only 13% of the participants reported that they have ever been exposed to anti-vaccination information or materials. 

### 3.2. Attitude and Perception towards All Vaccines and Measles Vaccine

Table 2 shows that the PACV’s items are categorized in three subscales: immunization behavior (items 1 and 2), perceived safety and efficacy (items 7–10), and general attitudes and trust (items 3–6 and 11–15). For immunization behavior, about 89 (17.8%) of the respondents have delayed having their child receive a shot for reasons other than illness or allergy. Data about the perception of safety and efficacy showed that 16.6% were (very) concerned that the measles vaccine might not be effective (item 10), 13% were (very) concerned that the vaccine might not be safe (item 9), and 19% were (very) concerned that their child might have serious side effects from the measles vaccination (item 8). Concerning the general attitude and trust, only 3.6% of the participants considered themselves as (very) hesitant about childhood measles shots. The vast majority of the participants (96.2%) reported that they trust the information they receive about measles shots, and they have trust in their child’s doctors (88%) (item 15).

### 3.3. Factors Predicting Measles Vaccine Hesitancy:

Table 3 shows statistically significant linear associations between parents’ total score on the PACV and their exposure to anti-vaccination information or materials (β = −0.478, *p*-value < 0.001), their perception about the effectiveness of measles vaccines (β = 0.093, *p*-value = 0.020), age of the mother (β = 0.112, *p*-value = 0.017), rank of their child (i.e., the one aged 2–3 years) (β = −0.116, *p*-value = 0.015), and total number of children who are aged less than 5 years within the family (β = 0.098, *p*-value = 0.013).

### 3.4. Reasons behind the Measles Vaccine Hesitancy (Open-Ended Question)

Fifty-nine parents (12%) who partially vaccinated or did not vaccinate their children with the measles vaccine justified their hesitancy for different reasons. Most of the cited reasons were related either directly or indirectly to access issues (i.e., availability of the measles vaccine and vaccinators’ attitude) as well as inconvenience, such as practical and social circumstances, which prevented timely attending of measles vaccination sessions. Categorized according to the 3Cs model the following main reasons were provided:

- ***Confidence***. Some parents were distrustful of the side effects and the vaccine dose that is provided. For example: *“I am concerned about the side effects of the vaccine, and I have fear of fever”.*
  *“Iam worried about the dose (i.e., injection)”.*
- ***Convenience***. Other parents stated that they had difficulties in accessing the vaccination service, uncooperative vaccine providers, and unavailability of vaccination material. For example:
  *“Firstly, I went to the center, and I did not easily access the vaccine because the number of children was not enough to open the vaccine vial. So, we delayed the first dose, and when my child took it, I considered that he should not take the second dose”.*
  *“I went many times, but I could not vaccinate her because of unavailability of the vaccine and the crowdedness”.*
  *“The vaccination is on certain days and I am busy”.*
  *“I lost the card, and when I went to the health center, they refused to vaccinate him with the last dose. I did not look for it and I did not go again”.*
- ***Complacency***. Some of the parents did not care very much about the vaccination or did not seem to perceive the problem of measles as very serious. Examples provided were as follows:
  *“I did not care for the second dose. There is no specific reason”.*
  *“Laziness and carelessness”.*
  *“I have no specific reasons”.*
- ***Other reasons.*** Parents also did not go for the vaccination because of private circumstances, social problems in the family, or health problems. Reasons provided were:
  *“I gave him the first dose, but I thought that the second dose is taken when the child is aged 5 years or when he enters the school. As we know that the first dose is given at the health center while the second dose is given by the campaign in home, and this is what happened with all our children”.*
  *“We had someone died and I forget to go again”.*
  *“We had social circumstances”.*
  *“I did not remember earlier, and when I remembered the time over/gone”.*
  *“I was ill at the time of the second dose”.*
  *“I was pregnant and I forgot”.*
  *“Absence of his mother because we were divorced”.*

## 4. Discussion

This study aimed to assess measles vaccine hesitancy and characterize its determinants among Sudanese parents in Omdurman in Khartoum State.

We found in this study that a significant proportion of participants (about 1 in 5 parents) have a risk perception of the measles vaccine (i.e., concerns about measles vaccine safety, efficacy, and the number of vaccines that the child received at the same session). Additionally, less than one-fifth of the respondents (18%) have ever delayed the measles vaccine for reasons other than illness or allergy. Given the high educational level of mothers (about 50% with a university degree) and the urban settings of the participants, these figures should be interpreted accordingly, although recent findings also detected vaccine hesitancy among high socioeconomic individuals in some LMICs, including Sudan [16,29]. Despite these findings contradicting the participants’ perception of the importance, safety, and effectiveness of measles vaccines, it is mirrored by the report from SAGE/WHO, which described vaccine-hesitant individuals as those who may refuse some vaccines but agree to others, delay vaccines, or accept vaccines but remain unsure in doing so [3]. Additionally, the latter finding is supported by similar studies that found parents are concerned about the side effects of the measles vaccine [36,37,38].

Remarkably, our findings revealed that parents who have ever been exposed to any anti-vaccination information or campaigns are more likely to be hesitant toward the measles vaccine. One of these anti-vaccine information messages circulating in the Sudanese community is that measles is a common household disease that can be contracted by every child, so topical or traditional medication can treat it [29]. Exposure to negative content and unreliable information about the vaccine from untrusted sources, which is known as “infodemic”, has been well established in recent years as a risk factor for vaccine hesitancy especially with the current pandemic of COVID-19 [39].

Perception of the effectiveness of measles vaccines (ranged from strongly agree to strongly disagree) was negatively associated with measles vaccines hesitancy. Similar findings from a recent study in Sudan [40] showed that pregnant women with low confidence towards vaccination were more likely to be involved in searching for additional vaccine information. Vaccination-related decisions of pregnant women in Italy were also found to be influenced by their exposure to information about vaccines [25].

Additionally, our findings showed that measles vaccine hesitancy increased with the increase in the age of the mother and the number of children. This may be attributed to the fact that Sudanese mothers are mostly the first persons responsible for the health and prevention of sickness of their children and family; therefore, larger families with many children under five years may increase the burden of mothers and may reduce the quality of care for the siblings. Some parents are hesitant towards vaccination when they have many children, because “they are afraid of the evil eye (i.e., they think that people will notice that they have many children in their house, so they think some of them will die)” [29]; therefore, they vaccinate some and leave the others unvaccinated. Additionally, Sudanese parents are not always aware of the importance of the “second dose”, especially if a mother feels that “a child is going strong at the age of 9 months” (i.e., the time of the first measles vaccine dose). Some of these findings are supported by studies from Malawi, Ethiopia, Kenya, and Tanzania, where parents are most likely to be hesitant and missing the opportunity to vaccinate with the second dose of measles vaccine when they have a larger sized family, increasing birth order, and increasing child age [22,23]. Fathers’ involvement in child health services has been well established as a factor associated with the improvement of decisions related to preventive health behaviors [41,42]. In light of these findings, we suggest fathers’ involvement may help reduce delays in vaccination in situations where the mother is unavailable because of different social reasons, such as traveling or caring for the other family members.

However, our findings showed that measles vaccine hesitancy was negatively associated with the rank of the child (who is aged 2–3 years). A recent study in Sudan showed that women who had the first pregnancy were actively searching for information about vaccines and immunization during their pregnancy; additionally, the same study showed that mothers who were low in confidence in the vaccine were more likely to search for such information [39]. In addition, some studies suggested that decision making about vaccination begins during pregnancy, especially among first-time mothers [43,44,45]. Not surprisingly, education level had no impact on measles vaccine hesitancy in our study. The latter finding is supported by another study conducted in five LMICs where education had no role in vaccine hesitancy among parents [46]. These findings suggest the importance of developing tailored vaccine communication strategies and interventions to increase confidence in vaccines and to reduce measles vaccine hesitancy among at-risk groups who are mostly exposed to negative information about the vaccine.

Interestingly, vaccination access-related issues were the common justifications behind hesitant parents’ decision to either delay, partially vaccinate, or refuse to vaccinate their children with measles vaccine. Given the open vial policy [10-dose vial] of measles vaccine in Sudan as well as in many LMICs, in addition to the lack of parental reminders [10,29,45], these results are not surprising. More research is needed to determine the optimal size of the measles vaccine vial in Sudan and its cost-effectiveness. At the program level, we suggest that an Expanded Program on Immunization set up parental reminders to address the long period between the vaccines generally and, in particular, for the 9th and 18th month measles’ doses.

Given the study’s findings, which showed the complexity of measles vaccine hesitancy in Sudan, we expect that the current evolving COVID-19 pandemic worldwide, and particularly in Sudan, where the vaccination services that provide routine vaccines including the measles vaccine have been disrupted for a while, along with reports of hesitancy towards COVID-19 vaccines in Sudan, will worsen the rate of uptake of measles vaccines in Sudan.

### Limitations

Findings should be interpreted within the socioeconomic and sociocultural context of this study. These include unintentional selection bias due to the sampling technique (consecutive sampling) to recruit the participants until 500 participants were reached from both districts. Additionally, half of the female participants (50%) reported that they had a university education. This figure is higher than the average rates for females (about 15% and 30% at the national and Khartoum State levels, respectively) [46]. Most of the participants were female; this may be attributed to the time when the interviews were conducted (i.e., men at work), which led to missing fathers’ perceptions, perspectives, and their role in making decisions about vaccination. However, we are not sure to what extent and in what direction these limitations might have biased our findings.

## 5. Conclusions

Measles vaccine hesitancy in Sudan is determined by various interrelated factors that range from individual to societal levels. Our findings underscored that a significant proportion of participants (about 1 in 5 parents) have a serious perception of the risk of the measles vaccination as well as practical and access issues related to measles vaccination sessions. As exposure to anti-vaccine information predicts measles vaccine hesitancy among parents, investment in vaccine communication will be a cost-effective intervention for measles vaccine acceptance and demand. In addition, changing the open vial policy for measles vaccine will improve access to measles vaccine in Sudan and, thus, increase vaccine coverage. Given the complexity of the current pandemic of the COVID-19 virus, research is needed for a further understanding of the impact of this pandemic on the trend of the uptake of measles vaccine in Sudan.

## Figures and Tables

**Table 1:** Sociodemographic characteristics of the respondents and their status of exposure to vaccination materials/campaigns.

		N	%
Residence of the respondent	Alsharafia	154	30.8
	Abo Saeed	346	69.2
Participants	Mother	436	87.2
	Father	23	4.6
	Others	41	8.2
Educational level of the mother	None	14	2.8
	Primary	62	12.6
	Secondary	173	34.6
	University	250	50.0
Mother’s employment	Housewife	373	74.6
	Employed	127	25.4
Income level (Self-ranking)	High	70	14.0
	Medium	395	79.0
	Low	35	7.0
Number of children < 5 years	1	222	44.4
	2	228	45.6
	3 and more	50	10.0
Rank of the child aged 2–3 years	1st	148	29.6
	2nd	126	25.2
	3rd	85	17.0
	4th and more	139	27.8
	Missing	2	0.4
Sex of the youngest child aged 2–3 years	Male	222	44.4
	Female	278	55.6
Measles vaccination status of the youngest child aged 2–3 years	Fully vaccinated	436	87.2
	Partially vaccinated	45	9.0
	Unvaccinated	14	2.8
	Don’t Know/Not sure	5	1.0
Exposure to any pro-vaccination materials/information	Exposed	387	77.4
	Not exposed	113	22.6
Exposure to any anti-vaccination materials/information	Exposed	65	13.0
	Not exposed	435	87.0

**Table 2:** Frequency distribution of the 15 PACV items (N = 500).

No.	PACV’s Items		N (%)
1	Have you ever delayed having your child get the measles vaccine for reasons other than illness or allergy?	Yes	89 (17.8)
		No	406 (81.2)
		I don’t know	5 (1.0)
2	Have you ever decided not to have your child get the measles vaccine for reasons other than illness or allergy?	Yes	11 (2.2)
		No	485 (97.0)
		I don’t know	4 (0.8)
3	How sure are you that following the recommended measles vaccine schedule is a good idea for your child?	0–5	19 (3.8)
		6–7	42 (8.4)
		8–10	439 (87.8)
4	Children get more shots of measles vaccine than are good for them.	Strongly agree	16 (3.2)
		Agree	21 (4.2)
		Not sure	18 (3.6)
		Disagree	294 (58.8)
		Strongly disagree	150 (30.0)
		Missing	1 (0.2)
5	I believe that measles is a severe disease.	Strongly agree	306 (61.2)
		Agree	174 (34.8)
		Not sure	6 (1.2)
		Disagree	14 (2.8)
6	It is better for my child to develop immunity by getting sick than to get a shot.	Strongly agree	26 (5.2)
		Agree	12 (2.4)
		Not sure	2 (0.4)
		Disagree	225 (45.0)
		Strongly disagree	235 (47.0)
7	It is better for children to get fewer vaccines at the same time.	Strongly agree	56 (11.2)
		Agree	58 (11.6)
		Not sure	14 (2.8)
		Disagree	254 (50.8)
		Strongly disagree	118 (23.6)
8	How concerned are you that your child might have a serious side effect from a shot of the measles vaccine?	Not at all concerned	208 (41.6)
		Not concerned	191 (38.2)
		Not sure	6 (1.2)
		Concerned	60 (12.0)
		Very concerned	35 (7.0)
9	How concerned are you that any one of the measles vaccine shots might not be safe?	Not at all concerned	253 (50.6)
		Not concerned	163 (32.6)
		Not sure	19 (3.8)
		Concerned	48 (9.6)
		Very concerned	17 (3.4)
10	How concerned are you that a shot of the measles vaccine might not prevent measles?	Not at all concerned	211 (42.2)
		Not concerned	188 (37.6)
		Not sure	17 (3.4)
		Concerned	62 (12.4)
		Very concerned	21 (4.2)
		Missing	1 (0.2)
11	If you had another infant today, would you want him/her to get all the recommended (measles) shots?	Yes	487 (97.4)
		No	9 (1.8)
		I don’t know	4 (0.8)
12	Overall, how hesitant about measles vaccine shots would you consider yourself to be?	Not at all hesitant	284 (56.8)
		Not hesitant	195 (39.0)
		Not sure	3 (0.6)
		Hesitant	12 (2.4)
		Very hesitant	6 (1.2)
13	I trust the information I receive about measles vaccine shots.	Strongly agree	242 (48.4)
		Agree	239 (47.8)
		Not sure	8 (1.6)
		Disagree	8 (1.6)
		Strongly disagree	2 (0.4)
		Missing	1 (0.2)
14	I am able to openly discuss my concerns about shots with my child’s doctor.	Strongly agree	272 (54.4)
		Agree	203 (40.6)
		Not sure	11 (2.2)
		Disagree	12 (2.4)
		Strongly disagree	2 (0.4)
15	All things considered, how much do you trust your child’s doctor?	0–5	25 (5.0)
		6–7	35 (7.0)
		8–10	440 (88.0)

**Table 3:** Result of the multivariate regression model for estimates of associations of parental measles vaccine hesitancy with potential predictors (N = 494).

	B	S.E	Beta	t	*p*-Value
Age of the mother	0.206	0.086	0.112	2.395	0.017 *
Number of children	1.505	0.606	0.098	2.481	0.013 *
The rank of the child	−0.786	0.320	−0.116	−2.451	0.015 *
Parental exposure to any pro-vaccination information/materials	−0.152	1.031	−0.006	−0.148	0.883
Parental exposure to any anti-vaccination information/materials	−15.152	1.267	−0.478	−11.958	0.000 *
Perception about the effectiveness of measles vaccines	1.716	0.734	0.093	2.339	0.020 *

* Statistically significant.
